# AMPA Receptor Within the Prelimbic Cortex Regulates Propofol‐Induced Locomotor Sensitization

**DOI:** 10.1111/adb.70078

**Published:** 2025-07-28

**Authors:** Chi Pan, Xinyu Mao, Yujie Jiang, Chenchen Jiang, Jiani Qiu, Yantong Zhang, Gang Chen, Mengting Xu, Jun Li, Binbin Wu

**Affiliations:** ^1^ Department of Anesthesiology, Perioperative and Pain Medicine The Second Affiliated Hospital and Yuying Children's Hospital of Wenzhou Medical University Wenzhou China; ^2^ Key Laboratory of Anesthesiology of Zhejiang Province The Second Affiliated Hospital and Yuying Children's Hospital of Wenzhou Medical University Wenzhou China; ^3^ Key Laboratory of Pediatric Anesthesiology, Ministry of Education Wenzhou Medical University Wenzhou China; ^4^ Clinical Research Unit The Second Affiliated Hospital and Yuying Children's Hospital of Wenzhou Medical University Wenzhou China

**Keywords:** AMPA receptor, locomotor sensitization, NMDA receptor, Prelimbic cortex, Propofol

## Abstract

Propofol is recognized as an addictive substance in both humans and animals. Increasing evidence suggests that the prelimbic cortex (PL) within the medial prefrontal cortex (mPFC), plays an important role in mediating drug addiction. In this study, we trained adult male Sprague–Dawley rats to establish a model of locomotor sensitization (LS). Moreover, optogenetic inhibition of glutamatergic neurons within the PL inhibited the LS of propofol, whereas optogenetic activation of glutamatergic neurons within the PL promoted the LS of propofol. This effect could be blocked by NBQX (a competitive AMPAR antagonist) pretreatment. Subsequently, a microinjection of NBQX (0.25‐1 μg/0.3 μL/site) or saline was administered into the bilateral PL to further examine the impact of AMPARs on the LS of propofol. We found that NBQX pretreatment significantly inhibited both the distance and activity in sensitized rats. The expressions of GluA1 and GluA2 subunits of AMPARs, phosphorylated NR1 subunit of NMDARs, D1Rs, phosphorylated ERK and phosphorylated CREB within mPFC were statistically significantly decreased after NBQX pretreatment, whereas, the expressions of total ERK, total CREB and total NR1 subunit remained unchanged. This evidence verifies the instrumental role of AMPARs within the PL in mediating the LS of propofol, and the NMDAR‐D1R/ERK/CREB signalling pathway may act as a potential mechanism.

## Graphical abstract

In this study, we trained adult male Sprague‐Dawley rats to establish a model of locomotor sensitization (LS). Moreover, optogenetic inhibition of glutamatergic neurons within the PL inhibited the LS of propofol, whereas optogenetic activation of glutamatergic neurons within the PL promoted the LS of propofol, and this effect could be blocked by NBQX (a competitive AMPAR antagonist) pretreatment. This evidence verifies the instrumental role of AMPARs within the PL in mediating the LS of propofol.

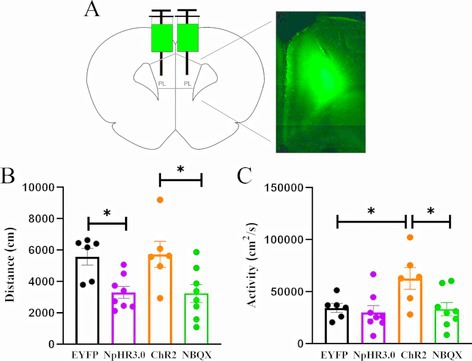

## 1 Introduction

Propofol, a widely used intravenous anaesthetic, has demonstrated to be an addictive substance in both humans and rodents [1, 2, 3]. Behavioural sensitization (BS), defined as the progressive increase in actions in psychomotor stimulant response following repeated drug exposure, is an effective model for studying behavioural plasticity [4]. The patterns of BS can be vary. In the present study, sensitization was observed based on the locomotor activity. Locomotor sensitization (LS) has been successfully established using various substances, including morphine, amphetamine and propofol [5, 6]. The perspective of sensitization posits that repeated use of drugs during the sensitization process can heighten responses to drug‐related cues, potentially leading to compulsive behaviours and relapse [5, 6, 7]. The dopamine pathway is critical in mediating drug dependence and psychiatric disorders and contributes to the development of LS [5]. The prefrontal cortex (PFC), regarded as a key center for higher cognitive function, has been shown to play an irreplaceable role in various psychiatric disorders, neurodegenerative diseases, memory and cognitive impairments [8, 9]. Previous studies have suggested that the medial prefrontal cortex (mPFC) is closely related to drug addiction, particularly to substances such as cocaine and alcohol [10, 11]. The mPFC comprises multiple types of neurons, including excitatory amino acid (glutamate) pyramidal neurons, cholinergic efferent neurons, cholinergic interneurons, and inhibitory gamma‐aminobutyric acid (GABA) interneurons. Functionally, the mPFC can be further subdivided into anterior cingulate, prelimbic (PL) and infralimbic (IL) cortices [8, 12]. The excitatory projection from the glutamate neurons in the PL to the nucleus accumbens (NAc) core (NAco) is regarded as a primary mechanism underlying cocaine‐induced BS [11]. However, the effect of PL on the development of LS‐related to propofol is yet to be explored. Glutamate, an important excitatory amino acid in the brain, plays a crucial role in the dopamine reward pathway [13]. Increasing evidence suggests that propofol exposure can enhance the expression of phospho‐calcium‐calmodulin (CaM)‐dependent protein kinase II (CaMKII) in both the mPFC and striatum through interaction with glutamate receptors, especially the α‐amino‐3‐hydroxy‐5‐methyl‐4‐isoxazolepropionic acid receptors (AMPARs) [14]. Our published research indicates that the projection from the basolateral nucleus of the amygdala (BLA) to the NAc shell (NAsh) regulates propofol self‐administration behaviour through AMPARs [15], which emphasizes the potential role of AMPARs in propofol addiction development.

The N‐methyl‐d‐aspartate receptor (NMDAR) is another type of ionotropic glutamate receptor, composed of the subunits of NR1 and NR2A‐D, each playing distinct roles in mediating the rat self‐administration of propofol and development of drug‐induced BS [16, 17]. It was reported that the gene expression of NR1 was promoted after repeated injections of cocaine [5]; while the expression of NR2B in striata is mainly associated with the maintenance of amphetamine‐induced BS [18]. Moreover, propofol causes acute LS via nitric oxide (NO)‐mediated mechanisms [6], which are closely related to the interaction between glutamate and NMDA receptors. Our previous research has reported that dopamine transmission within the mesolimbic system, especially involving the dopamine D1 receptor (D1R) and NMDAR‐D1R/extracellular signal‐regulated kinase (ERK)/cyclic‐AMP response element binding protein (CREB) signalling pathway in the NAc, is vital for regulating propofol self‐administration [15, 19, 20, 21]. Moreover, the activity of NMDAR can strongly influenced by AMPAR [22].

Nonetheless, the role of AMPARs in the PL in regulating propofol‐induced LS, as well as its potential mechanisms, remains unexplored. For instance, it is uncertain whether the subunits NR1 and NR2B of NMDARs are implicated in the central process of propofol‐induced LS and whether they operate through the NMDASR‐D1R/ERK/CREB signalling pathway. Consequently, we designed a study to investigate the role of AMPARs in the PL in regulating propofol‐induced LS using NBQX (a selective competitive antagonist of AMPARs) and optical stimulation. We also evaluated the impact of these interventions on the expression of NMDAR and D1R and their downstream ERK/CREB signalling pathway in the mPFC.

## 2 Materials and Methods

### 2.1 Animals

Adult male Sprague–Dawley rats, weighing approximately 250 g and aged 8 weeks, were acquired from the Experimental Animal Center of Wenzhou Medical University. The rats were housed in a temperature‐controlled room with a 12‐h light/dark cycle, maintained at 22–24 °C, and provided with free access to water and food. All the invasive operations were taken under sodium pentobarbital anaesthesia, and efforts were made to minimize the number of animals used and their sufferings. The study protocols adhered to the guidance of the Care and Use Committee of Wenzhou Medical University.

28 rats were designated for the groups treated with AAV‐CamkIIα‐EYFP + saline (EYFP group, *n* = 6), AAV‐CamkIIα‐eNpHR3.0‐EYFP + saline (NpHR3.0 group, *n* = 8), AAV‐CamkIIα‐ChR2(H134R)‐EYFP + saline (ChR2 group, n = 6) and AAV‐CamkIIα‐ChR2(H134R)‐EYFP + NBQX (NBQX group, *n* = 8). Additionally, another 29 rats were randomly assigned to the groups that receiving microinjections of NBQX (0.25, 0.5 or 1.0 μg/0.3 μL/site, n = 8) and vehicle (saline, n = 8) in the bilateral PL to test the effects of NBQX on propofol LS. Besides, another 24 rats were randomly disposed to the groups of NBQX (0.25, 0.5 or 1.0 μg/0.3 μL/site, *n* = 6) and vehicle (saline, n = 6), to test the impact of NBQX pretreatment in bilateral PL on locomotor activity.

### 2.2 Drugs

Propofol was obtained from Fresenius Kabi (10 mg/mL, Beijing, China); the dose of propofol used for the behavioural training was 20 mg/kg. NBQX (0.25, 0.5 and 1.0 μg/0.3 μL/site) (Sigma Aldrich, St‐Louis, USA), the selective antagonist of AMPARs, was dissolved in sterile saline (NS), and the doses of the agent applied in this study were determined based on our previous study [15, 21, 23].

### 2.3 Surgeries

**FIGURE 1:**
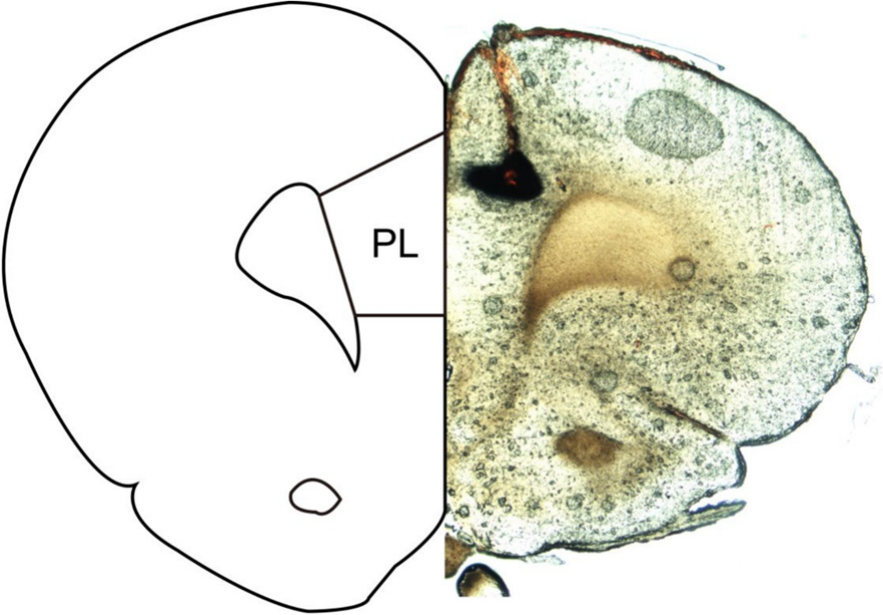
Schematic diagram showing ink injection site in the PL. Left: atlas, right: slice (50 μm).

The rats underwent implantation of cannulas under sodium pentobarbital anaesthesia (40 mg/kg ip) [23]. Microinjections in bilateral PL were performed through the implanted guide cannulas (20‐gauge, Small Parts Inc., USA) at the coordinate of AP + 3.4 mm, ML ± 0.5 mm, DV—3.4 mm (Figure 1).

### 2.4 Intra‐PL Microinjection Procedure

To assess the effects of NBQX on LS and locomotor activities, the rats were pretreated with either vehicle or NBQX (0.25, 0.5 and 1.0 μg/0.3 μL/site) in bilateral PL 30 min prior to the behavioural testing. Microinjections were conducted with a microinjection pump (MD‐1001, West Lafayette, USA) at the speed of 50 nL/min.

### 2.5 Stereotaxic Adeno‐Associated Viral Vector Injection in the PL

Adeno‐associated viral vectors (AAVs) are commonly used instruments in vivo studies. CamkIIα is a specific promoter of glutamatergic neurons and can link with AAV to enable glutamatergic neurons express the specific channel proteins of ChR2 or eNpHR3.0 on the surface of synapses.

To selectively target the excitatory neurons within the PL, the concentrated AAV (0.3 μL/side) (1 × 10^12^ infectious units/ml) encoding ChR2 or eNpHR3.0 were injected locally in the PL, driven by the CamkIIα promoter to transduce glutamatergic neurons. The AAV encoding EYFP served as a negative control. AAV‐CamkIIα‐‐EYFP, AAV‐CamkIIα‐ChR2(H134R)‐EYFP and AAV‐CamkIIα‐eNpHR3.0‐EYFP were acquired from Shumi Neuron Biotech Co. Ltd. (Wuhan, China). The injection of the AAV via 26‐gauge injector needles connected to a 1‐μl Hamilton syringe, and the microinjections were conducted under pentobarbital anaesthesia (40 mg/kg ip). Each microinjection lasted at least 10 min to allow the AAVs to spread throughout the PL. The accuracy of the AAV microinjections and local expression were accessed using a fluorescence microscope (Nikon, Japan). The rats trained for the experiments were 28 days after the virus injection.

### 2.6 Propofol‐Induced LS and Locomotor Activity Apparatus

**FIGURE 2:**
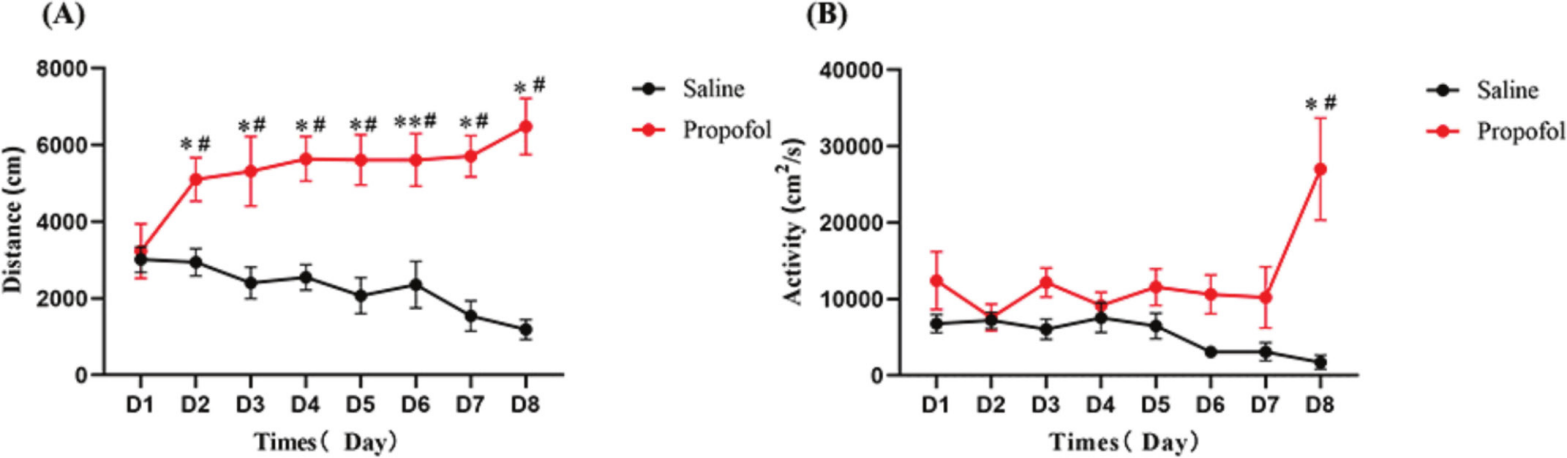
The model of propofol‐induced BS. (A) Effect of continuous intraperitoneal injection of propofol or saline on distance by rats (Mean ± SEM, *p < 0.05 compared with D1, #*p* < 0.05 compared with Saline group). (B) Effect of continuous intraperitoneal injection of propofol or saline on activity by rats (Mean ± SEM, *p < 0.05 compared with D1, #*p* < 0.05 compared with Saline group).

Adult male Sprague–Dawley rats were injected intraperitoneally with propofol (20 mg/kg) daily and trained for 7 consecutive days and half the dose of propofol was halved on the day 8 to establish a model of propofol LS. Compared with day 1, the distance and activity level of rats increased significantly, which indicated that rats treated with continuous injection of propofol produced LS, indicating the success of the propofol sensitization model (Figure 2). General locomotor activity was assessed using a unique motor monitoring device measuring 100 cm × 100 cm × 50 cm (Panlab, Barcelone, USA), equipped with an image tracking and processing system. Both the LS and locomotor activity behavioural procedures were tested automatically by the computers (SMART V3.0.06).

### 2.7 Propofol‐Induced LS Testing Under Optical Stimulation

Before the rats were placed in the behavioural testing area, the fibre optical cable was inserted into the guide cannula that implanted in the bilateral PL. The fibre optical cable was coupled to a laser generator, emitting 470‐nm light (AAV‐CamkIIα‐ChR2‐EYFP, blue light) or 589‐nm light (AAV‐CamkIIα‐eNpHR3.0‐EYFP, yellow light) to control power to the fibre tip. During the behavioural test, the rats received optical stimulation (900 s duration, 20 Hz, 25 ms pulse width, 50 mW output power). All the behavioural data were automatically analysed and recorded by the computers.

### 2.8 Locomotor Activity Test

After a 2‐h acclimation, NBQX (0.25, 0.5 and 1.0 μg/0.3 μL/site) was bilaterally microinjected into the PL to evaluate its impact on general activity. The rats were observed for 1 h (*n* = 6). The path length, mean speed of activity and the duration of activities were monitored by a digital camera positioned above the experimental box and recorded by the image tracking and processing system (SMART 3.0 system, Panlab, Harvard Apparatus, USA).

### 2.9 Western Blot Analysis

After the behavioural test, the brain was immediately removed following euthanasia by injection of sodium pentobarbital (200 mg/kg, ip). The mPFC was dissected from the brain, and then total protein was extracted. Bicinchoninic acid (BCA) protein assay kits (Thermo Fisher Scientific, Massachusetts, USA) were used to equipoise the concentration of the proteins. After boiling at 95 °C for 10 min, equal amounts of protein were loaded and separated by sodium dodecyl sulphate‐polyacrylamide gel electrophoresis (SDS‐PAGE). Then, the proteins were transferred to polyvinylidene fluoride (PVDF) membranes (NCM Biotech, Suzhou, China), and treated with NcmBlot Blocking Buffer (NCM Biotech, Suzhou, China) at room temperature for 20 min. After that, the bands were incubated overnight at 4 °C with primary antibodies (NR1, p‐NR1 (Ser896), NR2B, p‐NR2B (Tyr1472), p‐ERK and p‐CREB, rabbit, 1:1000, Cell Signalling Technology, Danvers, MA, USA; GluA1, GluA2, D1R, ERK and CREB, rabbit, 1:1000, Abmart, Shanghai, China; GAPDH, mouse, 1:10000, Proteintech, Chicago, USA) on a shaker. Afterward, the membranes were incubated with a secondary antibody (goat anti‐rabbit or goat anti‐mouse, 1:3000, Proteintech, Chicago, United States) diluted with tris‐buffered saline (TBST) at room temperature for 2 h. Protein bands were visualized with Enhanced Chemiluminescence (ECL) Kit (NCM Biotech, Suzhou, China), and scanned with the Image Quant LAS 4000 mini (GE Healthcare, Chicago, IL, USA).

### 2.10 Statistical Analysis

All data in this study were described as continuous variables and presented as the mean ± standard error of mean (SEM). The normality of data distribution was assessed prior to further analyses. For normally distributed data that met the assumption of homogeneity of variance, unpaired t‐test or one‐way analysis of variance (ANOVA) was applied for comparisons. Dunnett's post hoc test was used for multiple comparisons when ANOVA indicated significance. For data that were not normally distributed, the Kruskal‐Wallis test was utilized, followed by Dunn's post hoc analysis when significance was found in multiple comparisons. All statistical calculations were carried out using GraphPad Prism (version 6.0; GraphPad Software Inc. La Jolla, CA, USA) and SPSS (version 25.0; IBM Corp, Armonk, NY, USA), and a p‐value smaller than 0.05 was considered statistically significant.

## 3 Results

### 3.1 The Facilitation of Intra‐PL Microinjection of AAV‐CamkIIα‐ChR2(H134R)‐EYFP on the LS of Propofol can Be Inhibited by the Pretreatment With NBQX in Bilateral PL.

**FIGURE 3:**
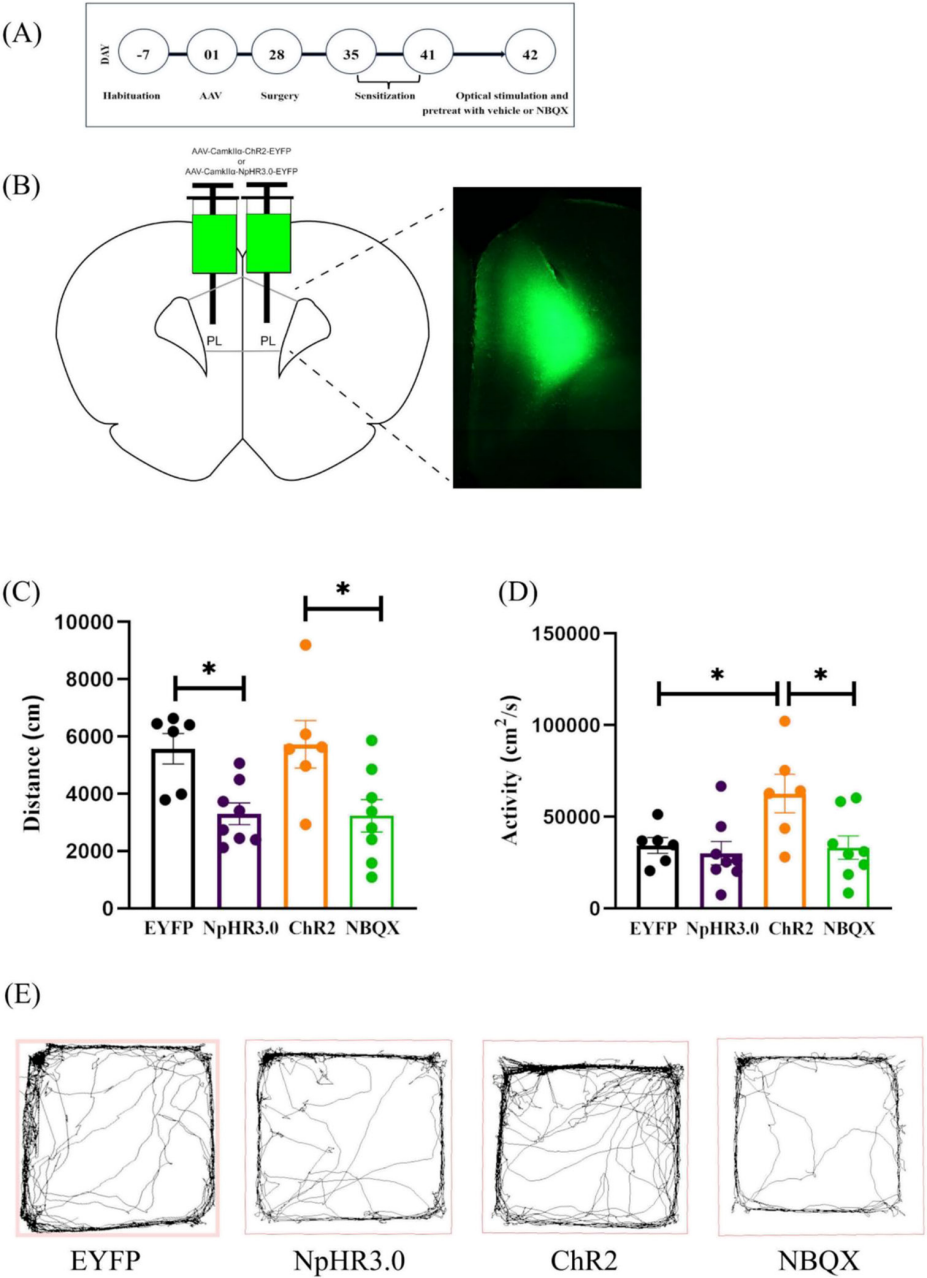
The timeline of experiment (A). The local expression of the AAV was examined under a fluorescence microscope. Left: atlas, right: slice (200 μm), the arrow indicates the location of PL (B). The behavioural results of EYFP, NpHR3.0, ChR2 and NBQX groups (C, distance; D, activity). Movement trajectory diagrams for all groups (E).

The local expression of the AAV was examined in the PL area (Figure 3B) after the transduction for 28 days. The behavioural results showed that compared with the EYFP group, the distance (Figure 3C, EYFP group: 5571 ± 537.3 cm; NpHR3.0 group: 3299 ± 377.8 cm) was significantly shorter in the NpHR3.0 group (*p* = 0.0207, Dunn), and the activity level (Figure 3D, EYFP group: 34409 ± 4318 cm^2^/s; NpHR3.0 group: 30182 ± 6384 cm^2^/s) had no significantly change (*p* > 0.05, Dunn). There was no significant change (*p* > 0.05, Dunn) in the distance (EYFP group: 5571 ± 537.3 cm; ChR2 group: 5727 ± 828.9 cm) but a significant increase (*p* = 0.0351, Dunn) in the activity (EYFP group: 34409 ± 4318 cm^2^/s; ChR2 group: 62701 ± 10 454 cm^2^/s) of the ChR2 group as compared to the EYFP group; compared with the ChR2 group, the NBQX group had a significantly shorter distance (ChR2 group: 5727 ± 828.9 cm; NBQX group: 3230 ± 569.1 cm) (*p* = 0.0244, unpaired t‐test), and significantly lower activity (ChR2 group: 62701 ± 10 454 cm^2^/s; NBQX group: 33202 ± 6413 cm^2^/s) (*p* = 0.0263, unpaired t‐test).

### 3.2 The LS of Propofol Was Inhibited by the Pretreatment of NBQX in Bilateral PL.

**FIGURE 4:**
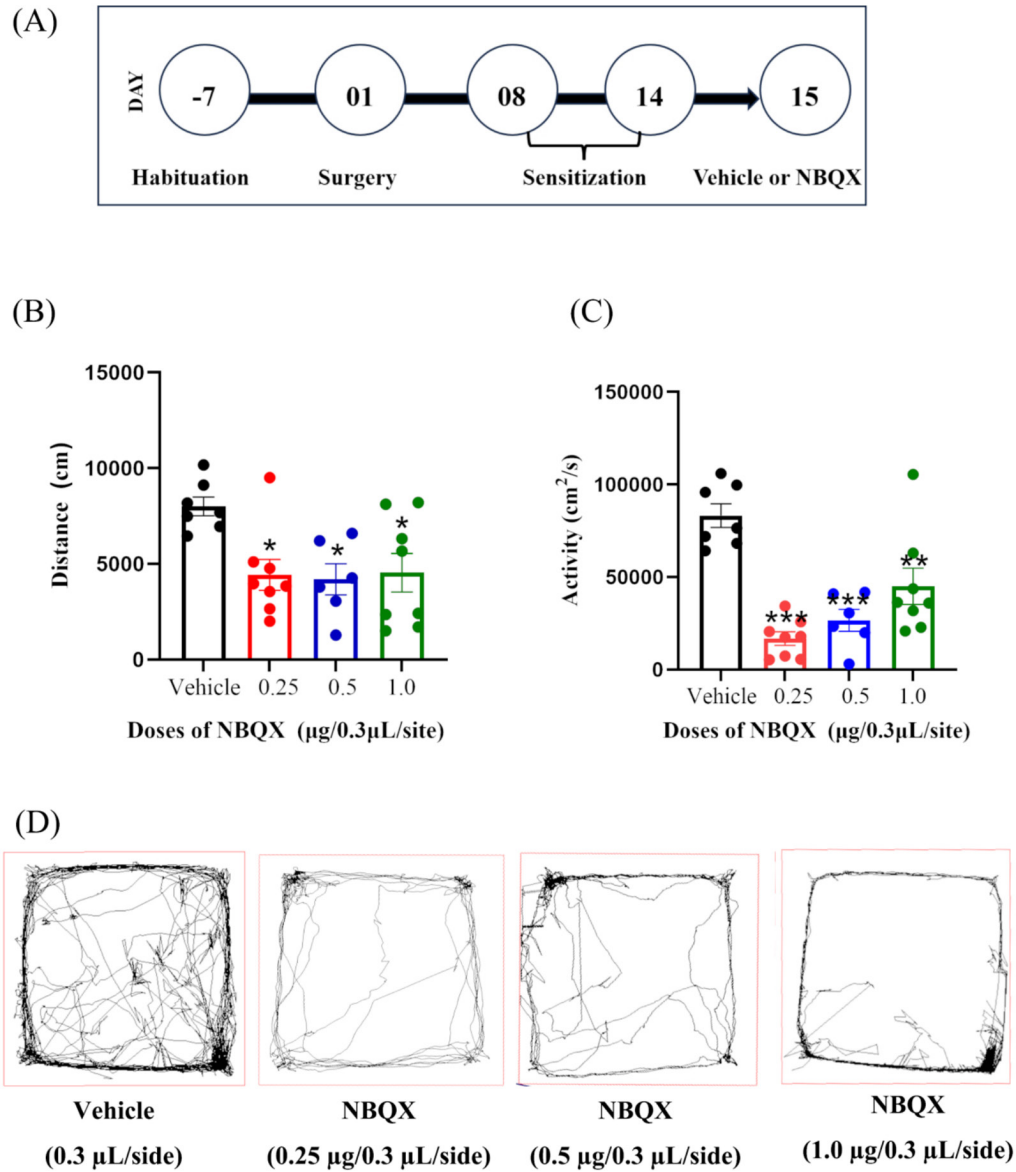
The schematic timeline of experiment (A). The pretreatment of NBQX in bilateral PL statistically significantly inhibited the distance (B) and activity (C) of propofol‐induced BS (vehicle group *n* = 7, 0.25 μg group *n* = 8, 0.5 μg group *n* = 6, 1.0 μg group n = 8). Movement trajectory diagrams for all behavioural experimental groups (D). One‐way ANOVA was applied for the normally distributed data, and dunnett‘s post hoc test for multiple comparisons. *p < 0.05, **p < 0.01, ***p < 0.001.

Both the distances and activities were statistically significantly reduced in the rats pretreated with NBQX (0.25, 0.5 and 1.0 μg/0.3 μL/site) in bilateral PL, compared to the vehicle group (Figure 4. distance, df = 3, F = 4.68, *p* = 0.01; activity, df = 3, F = 10.38, *p* = 0.002; vehicle group *n* = 7, 0.25 μg group *n* = 8, 0.5 μg group *n* = 6, 1.0 μg group n = 8).

### 3.3 The Expressions of GluA1 and GluA2 Subunits of AMPARs, and Phosphorylated NR1 and Phosphorylated NR2B Subunits of NMDARs Were Statistically Significantly Decreased, but the Expressions of Total NR1 and Total NR2B Subunits Remained Unchanged

**FIGURE 5:**
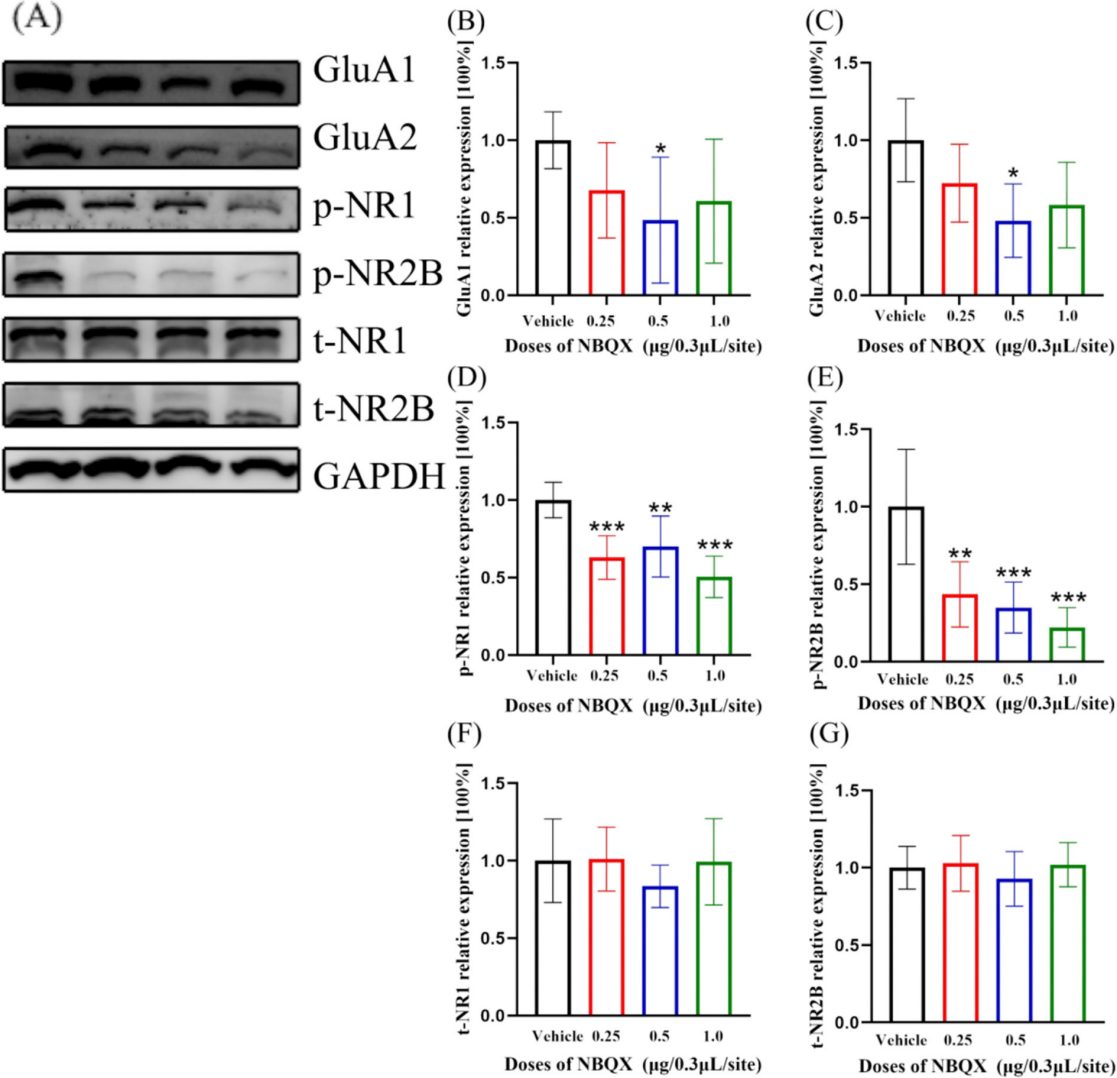
The expressions of GluA1 (B) and GluA2 (C) were statistically significantly decreased by the NBQX pretreatment at the dose of 0.5 μg/0.3 μL/site (GluA1, *p* = 0.021, n = 6; GluA2, *p* = 0.016, *n* = 5). The expressions of p‐NR1 (D) and p‐NR2B (E) were also statistically significantly decreased by NBQX pretreatment at the dose of 1.0 μg/0.3 μL/site (p‐NR1, *p* < 0.001, n = 6; p‐NR2B, *p* < 0.001, n = 6). In contrast, the expressions of t‐NR1 (F, *p* = 0.590, n = 5) and t‐NR2B (G, *p* = 0.698, n = 6) did not changed. One‐way ANOVA was applied for the normally distributed data, with Dunnett's post hoc test for multiple comparisons; otherwise, Kruskal–Wallis test with the Dunn's post hoc analysis was used. *p < 0.05, **p < 0.01, ***p < 0.001.

The expressions of GluA1 (Figure 5B, df = 3, H = 7.847, *p* = 0.049, *n* = 6, Kruskal–Wallis) and GluA2 (Figure 5C, df = 3, F = 3.802, *p* = 0.031, *n* = 5, one‐way ANOVA) subunits of AMPARs, and phosphorylated NR1 (Figure 5D, df = 3, F = 11.89, *p* < 0.001, *n* = 6, one‐way ANOVA) and phosphorylated NR2B (Figure 5E, df = 3, F = 12.59, *p* < 0.001, *n* = 6, one‐way ANOVA) subunits of NMDAR within mPFC were statistically significantly decreased, but the expressions of total NR1 (Figure 5F, df = 3, F = 0.66, *p* = 0.590, n = 5, one‐way ANOVA) and total NR2B (Figure 5G, df = 3, F = 0.48, *p* = 0.698, *n* = 6, one‐way ANOVA) subunits were not changed.

### 3.4 The Expressions of D1R, Phosphorylated ERK and Phosphorylated CREB Within mPFC Were Significantly Decreased by Pretreatment of NBQX in the PL, However, the Expressions of Total ERK and Total CREB Remained Unchanged

**FIGURE 6:**
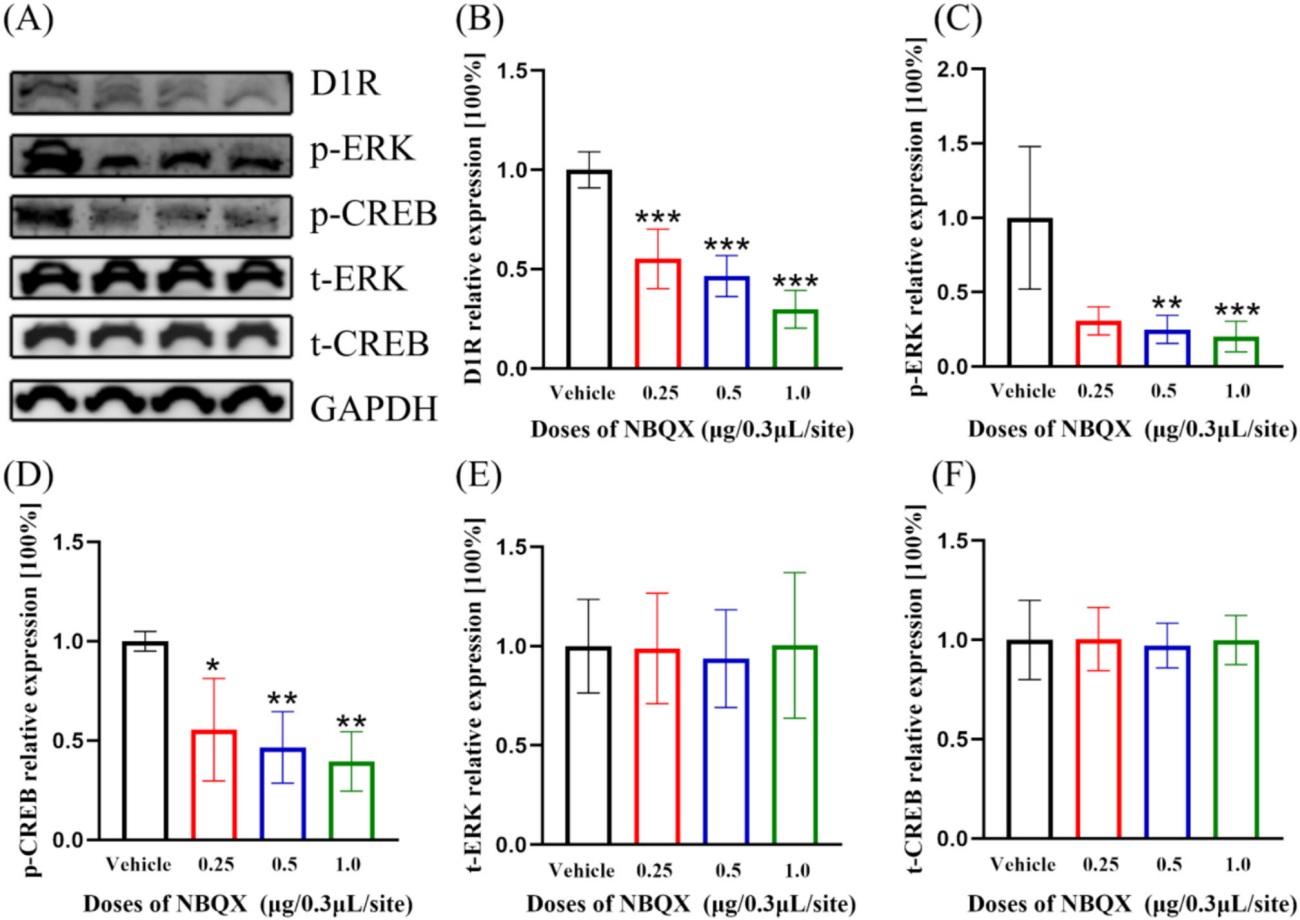
The expressions of D1R (B), p‐ERK (C) and p‐CREB (D) were statistically significantly decreased by NBQX pretreatment at the dose of 1.0 μg/0.3 μL/site (D1R, *p* < 0.001, n = 6; p‐ERK, *p* < 0.001, n = 6; p‐CREB, *p* = 0.003, n = 6). The expressions of t‐ERK (E, *p* = 0.976, n = 6) and t‐CREB (F, *p* = 0.982, n = 6) remained unchanged. One‐way ANOVA was applied for the normally distributed data, with Dunnett's post hoc test for multiple comparisons; otherwise, the data were analysed by Kruskal–Wallis test with the Dunn's post hoc analysis for multiple comparisons. *p < 0.05, **p < 0.01, ***p < 0.001.

The expressions of D1R (Figure 6B, df = 3, F = 42.85, *p* < 0.001, *n* = 6, one‐way ANOVA), phosphorylated ERK (Figure 6C, df = 3, H = 15.01, *p* = 0.002, *n* = 6, Kruskal–Wallis) and phosphorylated CREB (Figure 6D, df = 3, H = 13.50, *p* = 0.004, *n* = 6, Kruskal–Wallis) within mPFC were all statistically significantly reduced by the pretreatment of NBQX in bilateral PL, but the expressions of total ERK (Figure 6E, df = 3, F = 0.069, *p* = 0.976, n = 6, one‐way ANOVA) and total CREB (Figure 6F, df = 3, F = 0.057, *p* = 0.982, n = 6, one‐way ANOVA) did not changed.

### 3.5 The Pretreatment of NBQX in Bilateral PL Did Not Result in any Significant Alteration in Locomotor Activities

**FIGURE 7:**
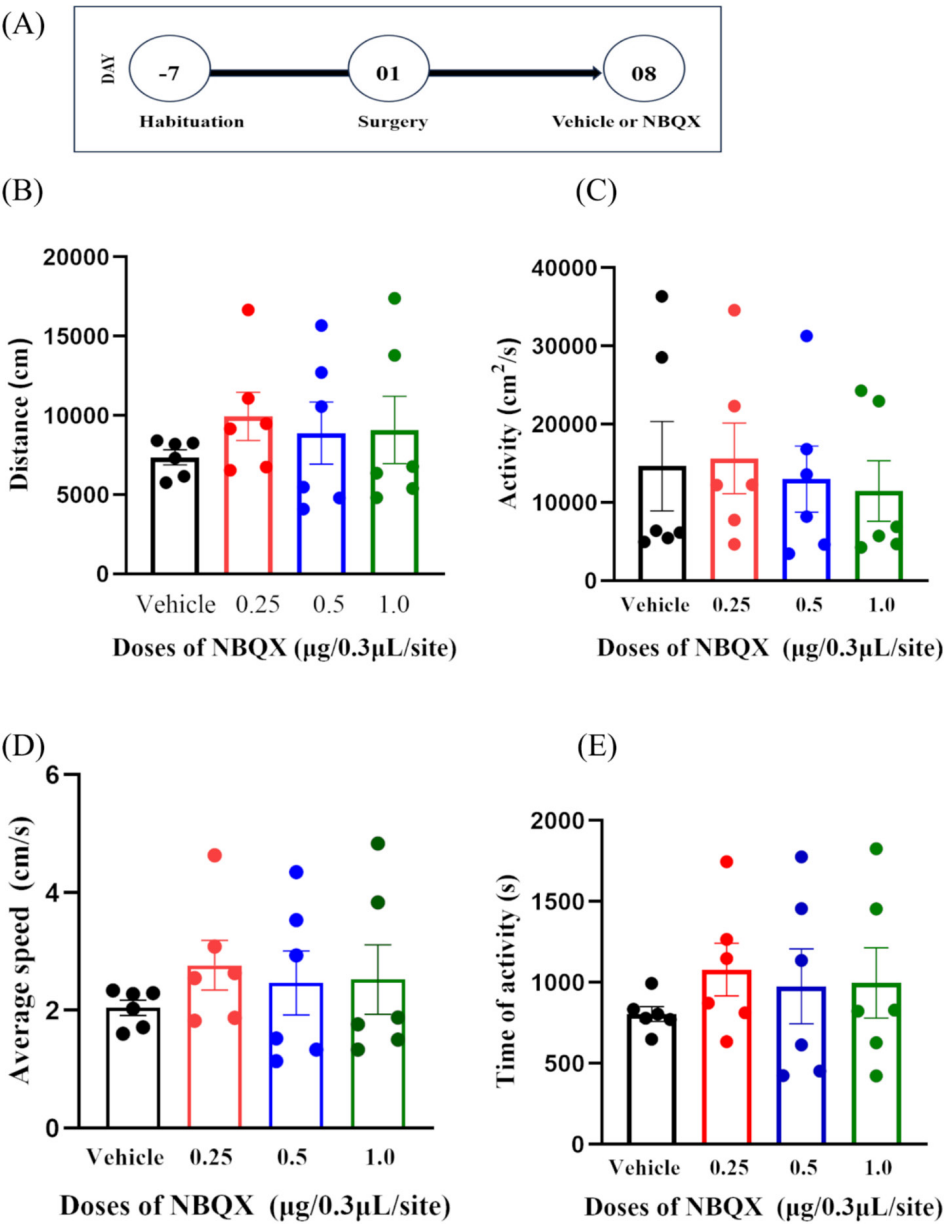
Schematic diagram of the locomotor activity testing. (A) The effects of NBQX pretreatment in bilateral PL on general activities. The results show that NBQX pretreatment did not statistically significantly affect locomotor activities, as measured by distance (B, *p* = 0.734, n = 6), activity (C, *p* = 0.854, n = 6), average speed (D, *p* = 0.734, n = 6) and the time of activity (E, *p* = 0.747, n = 6).

To determine the specificity of NBQX pretreatment in bilateral PL on LS, its impact on regular locomotor activities was investigated. The ANOVA analysis showed that NBQX pretreatment did not change the locomotor activities as accessed by the distance (Figure 7B, df = 3, F = 0.429, *p* = 0.734, n = 6, one‐way ANOVA), activity (Figure 7C, df = 3, H = 0.78, *p* = 0.854, n = 6, Kruskal–Wallis), mean speed (Figure 7D, df = 3, F = 0.429, *p* = 0.734, n = 6, one‐way ANOVA) or the time of activity (Figure 7E, df = 3, F = 0.410, *p* = 0.747, n = 6, one‐way ANOVA).

## 4 Discussion

In this study, the behavioural findings demonstrated that the LS of propofol in rats was inhibited following pretreatment with NBQX in the bilateral PL. This inhibition was accompanied by decreased expression of the subunits p‐NR1 and p‐NR2B, D1R, p‐ERK1/2, and p‐CREB in the mPFC, while the expression levels of t‐NR1, t‐NR2B, t‐ERK1/2, and t‐CREB remained unchanged. Furthermore, behavioural results from optical stimulation indicated that the facilitation of PL on the LS of propofol could be inhibited by antagonizing AMPARs in the PL. Collectively, these findings suggest that the PL plays a significant role in regulating propofol‐induced LS, potentially mediated through the AMPARs and NMDAR‐D1R/ERK/CREB signalling pathway. Additionally, general activities were not significantly affected by NBQX pretreatment.

The modelling of BS is chronic and adaptive, paired with enhancement in locomotor response or LS after repeated administration of psychostimulants [5]. LS, as one of the important manifestations of neuroadaptation about addiction, exhibits a strong dependence on substances [4, 5]. Recent studies have demonstrated that the development of LS is associated with increased vulnerability to drug reward [8] and relapses following the extinction of drug‐seeking behaviour [24]. These findings indicate that neuroadaptive changes are crucial to the transition from substance abuse to addiction. Among that, the release of glutamate and dopamine is thought to be necessary for the development of LS [4]. An increasing number of studies suggest that AMPARs are crucial in regulating glutamate neurotransmission and neuroplasticity across various pathophysiological conditions, including drug dependence [11]. AMPARs in the PL are involved in mediating behaviours associated with drug addiction, such as heroin self‐administration and extinction [25]. Both NMDARs and D1Rs in the PL have been demonstrated playing a key role in regulating cocaine memory reconsolidation [25, 26]. Nonetheless, the effects of AMPARs on drug associated behaviours can vary. Previous research has shown that intra‐lateral habenula microinjection of NBQX increases the behaviorally rewarding effects of a sub‐reward threshold dose of morphine [27]. However, multiple studies also support the finding that pretreatment of NBQX in animals can reduce drug‐seeking behaviours. For example, microinjection of NBQX into the NAsh prevents the morphine‐dependent rats from developing naloxone‐induced conditioned place aversions, and decreases the sensitivity to brain stimulation reward [28]. The inconsistencies in the results may stem from the different targeted brain regions used for pretreatment, as well as the distinct mechanisms underlying various forms of addiction. Our behavioural findings that pretreated with NBQX in bilateral PL inhibited the LS of propofol align with a previous study demonstrating that AMPAR trafficking is essential for modifying PL synaptic strength and facilitating memory erasure [29]. Moreover, the research has also found that dopaminergic signalling regulates AMPARs transport in the PL [30]. The retrieval of drug‐associated memory leads to a rapid and transient silencing of synaptic strength in the PL, which is mediated by the endocytosis of AMPARs from the membrane [30]. Interestingly, this central process is launched by dopamine in the PFC region and controlled by D1R signalling [30]. Dopamine receptors, especially the D1R, are considered participating in the modulation of AMPAR trafficking in the mPFC during long‐term potentiation, which is strongly associated with drug addiction [29].

GluA1 and GluA2 are prominent components of AMPARs, primarily located in the outer layers of the cerebral cortex. They paly significant roles in drug‐associated behaviours, such as cue‐induced seeking, incubation of craving, and sensitization [31, 32]. Their involvement in these processes highlights the importance of AMPARs in the neurobiological mechanisms underlying addiction and related behaviours. Recent studies have shown that GluA1 and GluA2 subunits can be locally synthesized and inserted into the surface of synapses, where they actively function in dendrites [33]. Our results indicate that pretreatment with NBQX in bilateral PL reduced the expression of GluA1 and GluA2, which are consistent with previous studies showed that NBQX's ability to reduce these subunits in primary neuron cells in vitro [34]. Furthermore, the translation of mRNAs related to the expressions of GluA1 and GluA2 subunits is regulated by the activation of the dopamine receptor in hippocampal neurons [30]. Thus, we propose that NBQX may diminish the interaction between AMPARs and NMDARs, and this process may work through the subunits of GluA1 and GluA2.

To further investigate the role of glutamatergic neurons within PL concerning propofol‐induced LS, we microinjected AAV‐CamkIIα‐ChR2‐EYFP or AAV‐CamkIIα‐eNpHR3.0‐EYFP into bilateral PL. We found that optical stimulated glutamatergic neurons within PL promoted the LS of propofol, and it can be attenuated by the pretreatment with NBQX in bilateral PL. These results align with previous evidence indicating that optogenetic manipulation of PL neurons promotes conditioned reward‐seeking behaviour after learning [34]. Thus, we postulate that the pretreated NBQX within PL may influence the function of PL neurons in drug related memory and synaptic plasticity, thereby reducing the sensitization to drugs in rats.

Modulation of NMDAR phosphorylation has been considered as one of the important mechanisms underlying NMDARs and DA receptor interactions after acute and chronic treatment by drug abuse. Activation of D1Rs enhances NMDARs mediated neural processing via various signalling cascades in both dorsal and ventral striatum [35]. These interactions significantly contribute to synaptic strengthening and drug reinforcement [23, 26]. The subunits of NR1 and NR2B are important components of NMDARs, and their significance in regulating drug dependence has been emphasized in numerous studies [16, 18]. For instance, knockdown the NR1 gene in central nucleus of the amygdala (CeA) neurons resulted in a reduction of morphine‐induced Fos protein labeling in the ventral bed nucleus of the stria terminalis (BNST) [36]. The NR2B subunit has also been linked to the maintenance of BS induced by amphetamine [18], and upregulating the of NR2B subunit in the NAc has been associated with cocaine sensitization [37]. In our study, we found that pretreatment with NBQX inhibited LS and was associated with decreased expressions of p‐NR1 (Ser897) and NR2B (Tyr1472) subunits, as well as D1R levels These findings are in agreement with a previous observation that D1R antagonists decreased the expressions of p‐NR1 (Ser897) and p‐NR2B (Tyr1472) subunits in the model of tumour cell implantation induced bone cancer pain in rats [38].

On the other hand, our findings indicate the pretreatment of NBQX within PL significantly affects NMDAR‐D1R/ERK/CREB signalling pathway. ERK signalling is an essential component of NMDAR signal transduction, as well as its downstream transcription factor CREB is also implicated in controlling long‐term synaptic plasticity, drug addiction and associative learning [39, 40]. In this study, we found that the expressions of p‐ERK and p‐CREB were significantly decreased. Several factors may explain these results. Firstly, the GLYX‐13 and ketamine have manifested profound and long‐lasting antidepressant effects by blocking NMDARs, an effect can be intercepted by NBQX pretreatment [41]. This result suggests that the AMPARs may fulfil its role in the neuronal network by regulating the functions of NMDARs. Besides, the D1Rs participated in regulating drug reinforcement memory and synaptic strength through their interaction with AMPARs, as previously noted [29]. The expression of D1Rs in the NAc can be statistically significantly decreased by NBQX pretreatment in propofol self‐administration rats [16]. Additionally, D1Rs are also essential for triggering cocaine‐induced ERK activation by potentiating NR2B‐containing NMDARs [42]. We propose that NBQX pretreatment may influence the expression of NR2B/ERK/CREB by disrupting the function of D1R and NMDARs. Furthermore, the evidence from electrophysiological experiments have demonstrated the major role of D1R/GluN1 complexes in the modulation of glutamatergic transmission and ERK‐dependent synaptic plasticity in D1R‐MSN specifically [38]. Thus, D1R/GluN1 complexes may serve as a bridge connecting AMPARs and NMDAR‐D1R/ERK/CREB signal transduction pathway.

In addition to BS, locomotor activity is considered as another important indicator of the psychopharmacological effects of a substance. Although studies have shown that acute propofol injection might affect the locomotive activity in rats and that this effect might be related to nitrergic [43], but it was also reported that NBQX showed no significant effects on general locomotor activity in the normal rats or the rat that received repeated amphetamine treatment [44]. These results are in agreement with our results that no significant difference was found in general locomotor activities after NBQX pretreatment in bilateral PL.

The limitations of this study should be acknowledged. In this study, we employed a protocol of continuous intraperitoneal injection of propofol to establish the model of LS. Although some research literature has reported that continuous injection can also successfully established [41], most literature adopts an intermittent dosing regimen [6]. This suggests that there may be multiple approaches to cause LS of propofol. While we found that the expressions of GluA1 and GluA2 subunits of AMPARs, p‐NR1 and p‐NR2B subunits of NMDARs, and D1Rs were significantly suppressed by NBQX pretreatment in the PL, the underlying mechanisms of their interaction remain unclear. In our future research, we plan to adopt subunit‐specific antagonists of AMPARs and NMDARs subunits and investigate the role of each subunit in the interactions with D1Rs in our following study. Other limitations include that the roles of AMPARs and NMDAR‐D1R/ERK/CREB signalling pathway in the PL‐to‐NAc circuit have not been explored, and the female rats were not included in the current study. All these questions would be addressed in future investigations.

## 5 Conclusions

In conclusion, this study highlights the vital role of AMPARs in the PL in modulating the LS of propofol. The interactions among AMPAR NMDARs and D1Rs, along with the downstream ERK/CREB signalling pathway likely underlie this process. These evidence supports the hypothesis that PL, especially the AMPARs within the PL, is critical for mediating the LS of propofol by NMDAR‐D1R/ERK/CREB signalling pathway.

## Author Contributions

WBB and LJ were responsible for the study concept, design and assisted the interpretation of findings; PC drafted the manuscript; PC, MXY and JYJ contributed to the acquisition of animal data. PC, CG, ZYT and XMT performed the data analysis; WBB, JCC and QJN helped the language improvement. All authors critically reviewed content and approved version for final publication.

## Ethics Statement

The animal studies protocols were approved by the Experimental Animal Center of Wenzhou Medical University with Protocol numbers: wydw2022–0725.

## Consent

Data/manuscript publication was approved by all authors.

## Conflicts of Interest

The authors declare no conflicts of interest.

## Data Availability

The data used to support the findings of this study are available from the first author upon request.

## References

[1] C. T. Walsh , “Propofol: Milk of Amnesia,” Cell 185, no. 25 (2022): 4861, 10.1016/j.cell.2022.11.018 . 36493757

[2] T. Uzbay and A. Shahzadi , “A Comprehensive Analysis of Propofol Abuse, Addiction and Neuropharmacological Aspects: An Updated Review,” Korean Journal of Anesthesiology 78, no. 2 (2025): 91–104, 10.4097/kja.24707 . 39676519 PMC12013994

[3] A. Shahzadi , T. Uskur , A. G. Akkan , B. Çevreli , and T. Uzbay , “Effects of Propofol on Conditioned Place Preference in Male Rats: Involvement of Nitrergic System,” American Journal of Drug and Alcohol Abuse 44, no. 2 (2018): 167–174, 10.1080/00952990.2017.1344681 . 28750179

[4] C. Delage , A. Morel , P. de Witt , et al., “BS to Psychostimulants and Opioids: What Is Known in Rodents and What Still Needs to Be Explored in Humans?,” Progress in Neuro‐Psychopharmacology & Biological Psychiatry. 127 (2023): 110824, 10.1016/j.pnpbp.2023.110824 . 37479108

[5] L. J. Vanderschuren , A. N. Schoffelmeer , A. H. Mulder , and T. J. De Vries , “Dopaminergic Mechanisms Mediating the Long‐Term Expression of Locomotor Sensitization Following Pre‐Exposure to Morphine or Amphetamine,” Psychopharmacology 143, no. 3 (1999): 244–253, 10.1007/s002130050943 . 10353426

[6] T. Uskur , A. Ö. Şenöz , B. Çevreli , A. Barlas , and T. Uzbay , “Propofol but not Dexmedetomidine Produce Locomotor Sensitization via Nitric Oxide in Rats,” Psychopharmacology (Berlin) 238, no. 2 (2021): 569–577, 10.1007/s00213-020-05707-5. 33169201

[7] T. E. Robinson and K. C. Berridge , “Addiction,” Annual Review of Psychology 54 (2003): 25–53, 10.1146/annurev.psych.54.101601.145237 . 12185211

[8] P. Vezina , “Sensitization of Midbrain Dopamine Neuron Reactivity and the Self‐Administration of Psychomotor Stimulant Drugs,” Neuroscience & Biobehavioral Reviews 27, no. 8 (2004): 827–839, 10.1016/j.neubiorev.2003.11.001 . 15019432

[9] P. L. Gabbott , T. A. Warner , P. R. Jays , et al., “Prefrontal Cortex in the Rat: Projections to Subcortical Autonomic, Motor, and Limbic Centers,” Journal of Comparative Neurology 492, no. 2 (2005): 145–177, 10.1002/cne.20738 . 16196030

[10] J. R. Mesa , E. Carter , Y. Padovan‐Hernandez , and L. A. Knackstedt , “Alcohol Consumption Modulates Prelimbic Cortex Response to Cocaine Following Sequential Cocaine and Alcohol Polysubstance use in the rat,” Frontiers in Pharmacology 14 (2023): 1132689. Published 2023 Mar 16, 10.3389/fphar.2023.1132689 . 37007027 PMC10060651

[11] J. Kwon , H. J. Kim , H. R. Lee , et al., “Rewiring of Prelimbic Inputs to the Nucleus Accumbens Core Underlies Cocaine‐Induced BS,” Biological Psychiatry 94, no. 5 (2023): 378–392, 10.1016/j.biopsych.2022.12.024 . 36906501

[12] P. G. Anastasiades and A. G. Carter , “Circuit Organization of the Rodent Medial Prefrontal Cortex,” Trends in Neurosciences 44, no. 7 (2021): 550–563, 10.1016/j.tins.2021.03.006 . 33972100 PMC8222144

[13] Y. Guo , H. L. Wang , X. H. Xiang , and Y. Zhao , “The Role of Glutamate and Its Receptors in Mesocorticolimbic Dopaminergic Regions in Opioid Addiction,” Neuroscience & Biobehavioral Reviews 33, no. 6 (2009): 864–873, 10.1016/j.neubiorev.2009.02.005 . 19428497

[14] R. Yasuda , Y. Hayashi , and J. W. Hell , “CaMKII: A Central Molecular Organizer of Synaptic Plasticity, Learning and Memory,” Nature Reviews Neurosciences 23, no. 11 (2022): 666–682, 10.1038/s41583-022-00624-2. 36056211

[15] Z. Dong , S. Xiang , C. Pan , et al., “The Excitatory Transmission From Basolateral Nuclues of Amygdala to Nucleus Accumbens Shell Regulates Propofol Self‐Administration Through AMPA Receptors,” Addiction Biology 28, no. 8 (2023): e13310, 10.1111/adb.13310 . 37500486

[16] H. X. Zhou and L. P. Wollmuth , “Advancing NMDA Receptor Physiology by Integrating Multiple Approaches,” Trends in Neurosciences 40, no. 3 (2017): 129–137, 10.1016/j.tins.2017.01.001 . 28187950 PMC5339030

[17] B. P. Chen , X. X. Huang , D. M. Dong , H. Wu , T. Q. Zhu , and B. F. Wang , “The Role of NMDA Receptors in Rat Propofol Self‐Administration,” BMC Anesthesiology 20, no. 1 (2020): 149, 10.1186/s12871-020-01056-0. 32539742 PMC7294660

[18] I. Brunk , C. Sanchis‐Segura , C. Blex , et al., “Amphetamine Regulates NR2B Expression in Go2α Knockout Mice and Thereby Sustains BS,” Journal of Neurochemistry 115, no. 1 (2010): 234–246, 10.1111/j.1471-4159.2010.06921.x. 20649838

[19] B. Wang , X. Yang , A. Sun , et al., “Extracellular Signal‐Regulated Kinase in Nucleus Accumbens Mediates Propofol Self‐Administration in Rats,” Neuroscience Bulletin 32, no. 6 (2016): 531–537, 10.1007/s12264-016-0066-1. 27783327 PMC5567483

[20] L. J. Wu , H. Toyoda , M. G. Zhao , et al., “Upregulation of Forebrain NMDA NR2B Receptors Contributes to BS After Inflammation,” Journal of Neuroscience 25, no. 48 (2005): 11107–11116, 10.1523/JNEUROSCI.1678-05.2005. 16319310 PMC6725642

[21] B. Wu , Y. Liang , Z. Dong , et al., “Glucocorticoid Receptor Mediated the Propofol Self‐Administration by Dopamine D1 Receptor in Nucleus Accumbens,” Neuroscience 328 (2016): 184–193, 10.1016/j.neuroscience.2016.04.029 . 27126557

[22] M. T. Stefanik , C. Sakas , D. Lee , and M. E. Wolf , “Ionotropic and Metabotropic Glutamate Receptors Regulate Protein Translation in co‐Cultured Nucleus Accumbens and Prefrontal Cortex Neurons,” Neuropharmacology 140 (2018): 62–75, 10.1016/j.neuropharm.2018.05.032 . 30077883 PMC7575416

[23] J. Li , C. Pan , B. Huang , et al., “NMDA Receptor Within Nucleus Accumbens Shell Regulates Propofol Self‐Administration Through D1R/ERK/CREB Signalling Pathway,” Addiction Biology 29, no. 5 (2024): e13401, 10.1111/adb.13401 . 38782631 PMC11116088

[24] T. J. De Vries , A. N. Schoffelmeer , R. Binnekade , et al., “Drug‐Induced Reinstatement of Heroin‐ and Cocaine‐Seeking Behavior Following Long‐Term Extinction Is Associated With Expression of Behavioral Sensitization,” European Journal of Neuroscience 10, no. 11 (1998): 3565–3571, 10.1046/j.1460-9568.1998.00368.x. 9824469

[25] B. M. Siemsen , A. R. Denton , J. Parrila‐Carrero , et al., “Heroin Self‐Administration and Extinction Increase Prelimbic Cortical Astrocyte‐Synapse Proximity and Alter Dendritic Spine Morphometrics That Are Reversed by N‐Acetylcysteine,” Cells 12, no. 14 (2023): 1812. Published 2023 Jul 8, 10.3390/cells12141812 . 37508477 PMC10378353

[26] R. E. See , “Dopamine D1 Receptor Antagonism in the Prelimbic Cortex Blocks the Reinstatement of Heroin‐Seeking in an Animal Model of Relapse,” International Journal of Neuropsychopharmacology 12, no. 3 (2009): 431–436, 10.1017/S1461145709000054 . 19236732 PMC2747000

[27] E. Amohashemi , P. Reisi , and H. Alaei , “Involvement of AMPA Receptors of Lateral Habenula in the Expression and Acquisition Phases of Morphine‐Induced Place Preference,” Brain Research 1798 (2023): 148150, 10.1016/j.brainres.2022.148150 . 36343725

[28] S. E. Russell , D. J. Puttick , A. M. Sawyer , et al., “Nucleus Accumbens AMPA Receptors Are Necessary for Morphine‐Withdrawal‐Induced Negative‐Affective States in Rats,” Journal of Neuroscience 36, no. 21 (2016): 5748–5762, 10.1523/JNEUROSCI.2875-12.2016. 27225765 PMC4879196

[29] X. Lv , J. Zhang , and T. F. Yuan , “Retrieval‐Extinction of Drug Memory Requires AMPA Receptor Trafficking,” Science Advances 8, no. 51 (2022): eadd6642, 10.1126/sciadv.add6642 . 36563160 PMC9788760

[30] W. B. Smith , S. R. Starck , R. W. Roberts , and E. M. Schuman , “Dopaminergic Stimulation of Local Protein Synthesis Enhances Surface Expression of GluR1 and Synaptic Transmission in Hippocampal Neurons,” Neuron 45, no. 5 (2005): 765–779, 10.1016/j.neuron.2005.01.015 . 15748851

[31] J. Terrier , C. Lüscher , and V. Pascoli , “Cell‐Type Specific Insertion of GluA2‐Lacking AMPARs With Cocaine Exposure Leading to Sensitization, cue‐Induced Seeking, and Incubation of Craving,” Neuropsychopharmacology 41, no. 7 (2016): 1779–1789, 10.1038/npp.2015.345 . 26585289 PMC4867110

[32] Y. Xia , G. S. Portugal , A. K. Fakira , et al., “Hippocampal GluA1‐Containing AMPA Receptors Mediate Context‐Dependent Sensitization to Morphine,” Journal of Neuroscience 31, no. 45 (2011): 16279–16291, 10.1523/JNEUROSCI.3835-11.2011. 22072679 PMC3235051

[33] W. Ju , W. Morishita , J. Tsui , et al., “Activity‐Dependent Regulation of Dendritic Synthesis and Trafficking of AMPA Receptors,” Nature Neuroscience 7, no. 3 (2004): 244–253, 10.1038/nn1189 . 14770185

[34] J. M. Otis , V. M. Namboodiri , A. M. Matan , et al., “Prefrontal Cortex Output Circuits Guide Reward Seeking Through Divergent Cue Encoding,” Nature 543, no. 7643 (2017): 103–107, 10.1038/nature21376 . 28225752 PMC5772935

[35] D. J. Surmeier , J. Ding , M. Day , Z. Wang , and W. Shen , “D1 and D2 Dopamine‐Receptor Modulation of Striatal Glutamatergic Signaling in Striatal Medium Spiny Neurons,” Trends in Neurosciences 30, no. 5 (2007): 228–235, 10.1016/j.tins.2007.03.008 . 17408758

[36] M. A. Beckerman and M. J. Glass , “The NMDA‐NR1 Receptor Subunit and the mu‐Opioid Receptor Are Expressed in Somatodendritic Compartments of Central Nucleus of the Amygdala Neurons Projecting to the bed Nucleus of the Stria Terminalis,” Experimental Neurology 234, no. 1 (2012): 112–126, 10.1016/j.expneurol.2011.12.034 . 22227057 PMC3329754

[37] H. Monyer , N. Burnashev , D. J. Laurie , B. Sakmann , and P. H. Seeburg , “Developmental and Regional Expression in the rat Brain and Functional Properties of Four NMDA Receptors,” Neuron 12, no. 3 (1994): 529–540, 10.1016/0896-6273(94)90210-0. 7512349

[38] E. Cahill , V. Pascoli , P. Trifilieff , et al., “D1R/GluN1 Complexes in the Striatum Integrate Dopamine and Glutamate Signalling to Control Synaptic Plasticity and Cocaine‐Induced Responses,” Molecular Psychiatry 19, no. 12 (2014): 1295–1304, 10.1038/mp.2014.73 . 25070539 PMC4255088

[39] N. T. Miningou Zobon , J. Jędrzejewska‐Szmek , and K. T. Blackwell , “Temporal Pattern and Synergy Influence Activity of ERK Signaling Pathways During L‐LTP Induction,” eLife 10 (2021): e64644 2021 Aug 10., 10.7554/eLife.64644 . 34374340 PMC8363267

[40] H. Zhai , Y. Li , X. Wang , and L. Lu , “Drug‐Induced Alterations in the Extracellular Signal‐Regulated Kinase (ERK) Signalling Pathway: Implications for Reinforcement and Reinstatement,” Cellular and Molecular Neurobiology 28, no. 2 (2008): 157–172, 10.1007/s10571-007-9240-3. 18041576 PMC11515050

[41] Y. A. Blednov , A. Da Costa , S. Mason , et al., “Apremilast‐Induced Increases in Acute Ethanol Intoxication and Decreases in Ethanol Drinking in Mice Involve PKA Phosphorylation of GABAA β3 Subunits,” Neuropharmacology 220 (2022): 109255, 10.1016/j.neuropharm.2022.109255 . 36152689 PMC9810330

[42] J. Burgdorf , X. L. Zhang , K. L. Nicholson , et al., “GLYX‐13, a NMDA Receptor glycine‐Site Functional Partial Agonist, Induces Antidepressant‐Like Effects Without Ketamine‐Like Side Effects,” Neuropsychopharmacology 38, no. 5 (2013): 729–742, 10.1038/npp.2012.246 . 23303054 PMC3671991

[43] A. H. Tezcan , A. Özçetin , O. Özlü , B. Çevreli , and T. Uzbay , “Locomotor Stimulation by Acute Propofol Administration in Rats: Role of the Nitrergic System,” Pharmacological Reports 67, no. 5 (2015): 980–985, 10.1016/j.pharep.2015.03.003 . 26398394

[44] Y. Li , A. J. Vartanian , F. J. White , C. J. Xue , and M. E. Wolf , “Effects of the AMPA Receptor Antagonist NBQX on the Development and Expression of Behavioral Sensitization to Cocaine and Amphetamine,” Psychopharmacology 134, no. 3 (1997): 266–276, 10.1007/s002130050449 . 9438676

